# Antimicrobial resistance in leprosy: results of the first prospective open survey conducted by a WHO surveillance network for the period 2009–15

**DOI:** 10.1016/j.cmi.2018.02.022

**Published:** 2018-12

**Authors:** E. Cambau, P. Saunderson, M. Matsuoka, S.T. Cole, M. Kai, P. Suffys, P.S. Rosa, D. Williams, U.D. Gupta, M. Lavania, N. Cardona-Castro, Y. Miyamoto, D. Hagge, A. Srikantam, W. Hongseng, A. Indropo, V. Vissa, R.C. Johnson, B. Cauchoix, V.K. Pannikar, E.A.W.D. Cooreman, V.R.R. Pemmaraju, L. Gillini, WHO surveillance network of antimicrobial resistance in leprosy, A. Kriswamati, Abdul Rahim Al-Samie, Ahamed Issoufou, Alexandre Tiendrebeogo, AmriMiraju Kingalu, Andriamira Randrianantoandro, Anil Kumar, Aurelie Chauffour, Aye Aye Win, Basudev Pandey, C.M. Agrawal, Christiana Widaningrum, Christine Schmotzer, Christophe Kafando, Chuda Mani Bhandari, Cynthia Sema, D.S. Vidanagama, David M. Scollard, Demmissew Beyene, Eliane Faria Morelo, Elizabeth Dizaneh Kassa, Enerantien Benoit Ramarolahy, Eric Claco, Ernesto ES. Villalon, Famoussa Sidibe, Fatoumata Sakho, Fomba Abdoulaye, Francisco F. Guilengue, Fransesca Gajete, Gadde Rajan Babu, Gado Moussa, Garib Das Thakur, Gemma Cabanos, Gouressi Sock, Greame Clugston, Hany Zaidy, Haruo Watanabe, Herman Joseph Kawuma, Irene Balenton Mallari, Isabella Maria Bernandes Goulart, Issoufou Ahamed, J. Subbanna, Jean Gabin Houzeo, Jean Norbert Mputu Luengu, Jeanne Bertolli, Jonathan Lloyd-Owen, Jorge Matheu, José Pereira Brunelli, Juan Camilo Beltran Alzate, Kapil Dev Neupane, Katsunori Osuga, Kazuko Yamaguchi, Khalid Azam, Khin Maung Lin, Kodia Momoudu, Kyaw Kyaw, Landry Bide, Le Huu Doanh, Ley Huyen My, Mahesh Shah, Mamadou Kodio, Mamadou Sidibe, Mannam Ebenezer, Maria Aparecida de Faria Grossi, Marivic F. Balagon, Marlience Canlonon, Masahiko Makino, Maung Maung Htoo, Md Jamsheed Ahmed, Mintsey-mi-Makuth Nadine, Florenda Orcullo Roferos, Hana Krismawati, Mya Thida, Myo Thet Htoon, K.D. Neupane, Nhu Ha Nguyen Phuc, NguyenThi Hai Van, Ngyuen Phuc Nhu Hai, Norisha Ishii, Oke Soe, Olga Amiel, Omar Tossou, Ousmane Konare, P.L. Joshi, P.V. Ranganadha Rao, Padebettu Krishnamurthy, Patrick J. Brennan, Phillipe Busso, Rajesh Bhatia, Mala Rakoto Andrianarivelo, D.R. Ramdas, Raoul Chabi, Renato Gusmao, Rita DjupuriIzwardy, Rosa Castalia Franca Riberio Soares, Rupendra Jhadav, Samira Buhrer, Sang-Nae Ray Cho, Shen Jianping, Shinzo Lzumi, Sumana Barua, Sundeep Chaitanya, Sylvestre Marie Roget Tiendrebeogo, Tan Hau Khang, Thomas P. Gillis, Toru Mori, V. Vijayalakshmi, Vedastus Deusdedit Kamara, Wang Wei, W.Cairn S. Smith, Wei Li, Woojin Lew, Yasin Al-Qubati, Yasuhiko Suzuki, Yoshio Nanba

**Affiliations:** 1) Université Paris Diderot, UMR 1137 IAME Inserm, APHP-Lariboisière, APHP-Pitie-Salpêtrière, Centre de Référence des Mycobactéries et de la résistance des mycobactéries aux antituberculeux, Paris, France; 2) American Leprosy Missions, Greenville, SC, USA; 3) Leprosy Research Centre, National Institute of Infectious Diseases, Tokyo, Japan; 4) Global Health Institute, Ecole Polytechnique Fédérale de Lausanne, Switzerland; 5) Fondation Raoul Follereau, Paris, France; 6) Instituto Oswaldo Cruz, Rio de Janeiro, Brazil; 7) Instituto Lauro de Souza Lima, Sao Paulo, Brazil; 8) National Hansen's Disease Programs, Baton Rouge, USA; 9) National JALMA Institute of Leprosy & Other Mycobacterial Diseases, Agra, India; 10) Stanley Browne Laboratory, TLM Community Hospital, Delhi, India; 11) Institute Colombiano de Medicina Tropical, Sabaneta, Antioquia, Colombia; 12) Mycobacterial Research Laboratories, Anandaban Hospital, Kathmandu, Nepal; 13) Lepra Blue Peter Public Health and Research Centre, Hyderabad, India; 14) Institute of Dermatology, Chinese Academy of Medical Sciences, National Center for STD and Leprosy Control, China CDC, China; 15) Airlangga University, Surabaya, Indonesia; 16) Department of Microbiology, Immunology, and Pathology, Colorado State University, Fort Collins, CO, USA; 17) Global Leprosy Programme, WHO Regional Office for South-East Asia, New Delhi, India

**Keywords:** Antibiotic, Dapsone, *Mycobacterium leprae*, Ofloxacin, Resistance, Rifampicin

## Abstract

**Objectives:**

Antimicrobial resistance (AMR) is a priority for surveillance in bacterial infections. For leprosy, AMR has not been assessed because *Mycobacterium leprae* does not grow *in vitro*. We aim to obtain AMR data using molecular detection of resistance genes and to conduct a prospective open survey of resistance to antileprosy drugs in countries where leprosy is endemic through a WHO surveillance network.

**Methods:**

From 2009 to 2015, multi-bacillary leprosy cases at sentinel sites of 19 countries were studied for resistance to rifampicin, dapsone and ofloxacin by PCR sequencing of the drug-resistance-determining regions of the genes *rpoB*, *folP1* and *gyrA*.

**Results:**

Among 1932 (1143 relapse and 789 new) cases studied, 154 (8.0%) *M. leprae* strains were found with mutations conferring resistance showing 182 resistance traits (74 for rifampicin, 87 for dapsone and 21 for ofloxacin). Twenty cases showed rifampicin and dapsone resistance, four showed ofloxacin and dapsone resistance, but no cases were resistant to rifampicin and ofloxacin. Rifampicin resistance was observed among relapse (58/1143, 5.1%) and new (16/789, 2.0%) cases in 12 countries. India, Brazil and Colombia reported more than five rifampicin-resistant cases.

**Conclusions:**

This is the first study reporting global data on AMR in leprosy. Rifampicin resistance emerged, stressing the need for expansion of surveillance. This is also a call for vigilance on the global use of antimicrobial agents, because ofloxacin resistance probably developed in relation to the general intake of antibiotics for other infections as it is not part of the multidrug combination used to treat leprosy.

## Graphical abstract

**Figure:**
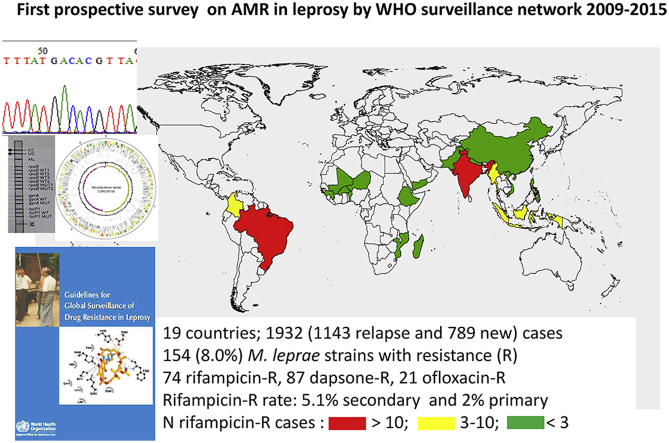

## Introduction

As treatment of leprosy and tuberculosis, the two main mycobacterial diseases, progressed during the 1950s and 1960s, the development of drug resistance was recognized as an obstacle to case management and control [1], [2], [3]. Resistance to dapsone, the first effective antileprosy drug, appeared in parallel with the emergence of resistance to streptomycin, the first antituberculosis agent [2]. To prevent further drug-resistance development, the treatment of both diseases was standardized with a combination of antibiotics. For tuberculosis, we know that this policy was only partially successful because multidrug resistance is unfortunately common and represents a major constraint on the control of tuberculosis [4]. For leprosy, to date, there have been no structured data available on resistance.

Dapsone resistance was evident as clinical failure on long-term monotherapy, but it was detected through laboratory tests only with the development of the mouse foot-pad model, because *Mycobacterium leprae* cannot be grown on any artificial media [5]. By 1981, dapsone resistance was widespread and rifampicin-resistant cases had emerged, which induced WHO to standardize multidrug therapy (MDT) for leprosy by combining dapsone with rifampicin for all cases, plus clofazimine for multi-bacillary cases [1], [6], [7]. MDT remains the recommended regimen for treating leprosy [8]. Although relapses in patients treated with MDT are rare [9], multiple resistance was eventually reported in different regions of the world, with the description of *M. leprae* strains resistant to dapsone, rifampicin and ofloxacin concomitant with treatment failure [10], [11].

The mouse foot-pad model was, until recently, the only method to test *M. leprae* for resistance to antimicrobial agents. This method is cumbersome, time-consuming (results are available only after 6–12 months), expensive and requires highly skilled laboratory staff [12]. For this reason, very little testing for resistance was done and only occasional cases of resistant leprosy were reported [1], [2], [13]. With the development of PCR and DNA sequencing, genetic markers of drug resistance in leprosy were identified for rifampicin, dapsone and ofloxacin (proxy for fluoroquinolones) [10], [14], [15], allowing results to become available within 1 day and at a lower cost. The samples for PCR testing were smear-positive skin-slit smear or skin biopsies, with sufficient bacillary load to allow *M. leprae* DNA to be amplified reproducibly [15], [16], [17], [18], [19]. No similar test is available for clofazimine because its mode of action and resistance are still unclear.

In view of the fact that MDT was introduced in the 1980s, and that no information was obtained on resistance rates, the WHO Global Leprosy Programme established in 2008 a surveillance network of laboratories able to perform molecular detection of resistance in leprosy. The primary objective was to monitor the possible emergence of resistance to rifampicin, the major antibiotic for leprosy therapy within MDT. Secondary objectives were to investigate also dapsone resistance, which is the rifampicin companion drug, and also ofloxacin resistance since fluoroquinolones are the second-line antibiotics recommended in case of rifampicin resistance and for cases with intolerance. This paper presents the results obtained to date by the network, orientating future antimicrobial resistance surveillance programmes.

## Materials and methods

### Patients

Patients with leprosy symptoms [20] were included and samples were collected before treatment started. After consent, patients were sampled by slit-skin smear or punch biopsy in active skin lesions. Inclusion criteria were multi-bacillary leprosy that was positive for bacteriological examination [21]. From 2009 onwards, we included relapse cases, defined as the appearance of new skin lesions and/or an increase in the bacteriological index of two or more units at any single site compared with the bacteriological index at the same site at a previous examination, at any time after the completion of a full course of treatment, after excluding leprosy reactions [21]. Since 2010, new cases were also included to allow the surveillance of primary resistance.

Each country applied the protocol under their good practices and ethics rules in clinics. Only aggregated data were collected and analysed by WHO and authors.

### Network composition and management

The network started in 2008 with six countries where *M. leprae* is endemic (Brazil, China, Colombia, India, Myanmar and Vietnam), and subsequently a total of 19 countries participated in the sentinel surveillance network (countries are listed in Table 1). Each country chose one or more ‘sentinel sites’ able to follow the protocol of antimicrobial resistance (AMR) surveillance. These sites were supported by governments through national leprosy programmes, often with the help of non-governmental organizations and international expert laboratories [21], [22].

**Table 1:** Surveillance of rifampicin resistance and number of rifampicin-resistant cases reported by country from 2009 to 2015

Country (WHO region)	Total leprosy cases			Relapse cases			New cases		
	Total	Number of Rif-R cases	Resistance rate (%)	Total	Number of Rif-R cases	Resistance rate (%)	Total	Number of Rif-R cases	Resistance rate (%)
Benin (AFR)	83	1	1.2	—	—	—	83	1	1.2
Brazil (AMR)	353	32	9.1	321	27	8.4	32	5	15.6
Burkina Faso (AFR)	2	0	0*	2	0	0*	—	—	—
China (WPR)	125	1	0.8	70	1	1.4	55	0	0
Colombia (AMR)	37	9	24.3	37	9	24.3	—	—	—
Ethiopia (AFR)	28	0	0	1	0	0*	27	0	0
Guinea (AFR)	23	1	4.3	1	0	0*	22	1	4.5
India (SEAR)	382	18	4.7	284	10	3.9	98	8	8.2
Indonesia (WPR)	91	3	3.3	91	3	3.3			—
Madagascar (AFR)	118	1	0.8	13	0	0*	105	1	0.9
Mali (AFR)	135	0	0	20	0	0*	115	0	0
Mozambique (AFR)	5	1	20.0*	5	1	20.0*	—	—	—
Myanmar (SEAR)	139	4	2.9	139	4	2.9	—	—	—
Nepal (SEAR)	69	1	1.4	56	1	1.8	13	0	0*
Niger (AFR)	47	1	2.1	12	1	8.3*	35	0	0
Pakistan (SEAR)	9	0	0*	9	0	0*	—	—	—
Philippines (WPR)	183	0	0	43	0	0	140	0	0
Vietnam (WPR)	86	0	0	28	0	0	58	0	0
Yemen (EMR)	17	1	5.9	11	1	9.1*	6	0	0*
Total	1932	74	3.8	1143	58	5.1	789	16	2.0

Abbreviations: AFR, African region; AMR, American region; EMR, Eastern Mediterranean region; SEAR, South-East Asian region; WPR, Western Pacific region.

—, not tested.

Asterisks indicate that reliable rates of resistance cannot be given because only a small number of cases were tested.

Eight network meetings were organized jointly by WHO and members of the International Federation of Anti-Leprosy Associations, inviting reference laboratories and national programme managers to collect and discuss data [22]. Early results were published in the WHO Weekly Epidemiological Record in 2010 and 2011 and in national reports [23], [24], [25], [26].

### Reference laboratories and molecular testing for drug resistance

Molecular testing was performed in ten national laboratories of endemic countries (three in India, five in Brazil, one in Colombia and one in China) and in four international expert laboratories (USA, France, Switzerland, Japan) for countries where testing could not be implemented nationally. Molecular testing for drug resistance involved the detection of mutations in defined genomic loci (drug-resistance-determining regions): *folP1* for dapsone resistance, *rpoB* for rifampicin and *gyrA* for ofloxacin. Each gene's drug-resistance-determining region contains the specific mutations associated with resistance in *M. leprae* in the mouse foot-pad method (described in the Supplementary material, Fig. S1) [27], [28].

A guide standardizing the protocol with details of basic techniques, such as PCR and sequencing, was published by WHO in 2009 [21]. Other techniques, such as automated PCR sequencing [29], micro-array [30], DNA strip [31], high-resolution melt analysis [32] and whole genome sequencing [33], which have been validated with regard to basic PCR sequencing and to mouse footpad, were then used from 2009 onwards according to each laboratory's expertise. Quality control panels (four *M. leprae* suspensions from positive mouse footpads harbouring wild-type or mutated genes) were prepared by the Baton Rouge and Tokyo expert laboratories and sent to all laboratories. Results showed 80% sensitivity and 100% specificity in the detection of *M. leprae* DNA, and 100% sensitivity and 99% specificity were observed for mutation detection.

### Dissemination of results and analysis

Resistance results were sent back from laboratories to programme managers and clinicians. Each year and at each surveillance meeting, data were collected and summarized as aggregated data. For the 2016 surveillance meeting, standard Excel tables were developed including the following variables: new or relapse case, presence or absence of mutations associated with resistance to dapsone, ofloxacin or rifampicin. Mutations that have not yet been associated with resistance were mentioned but were not counted as AMR cases. The tables were resubmitted to the laboratories for further verification of the results under the coordination of the WHO Global Leprosy Programme.

## Results

### Assessment of rifampicin resistance in leprosy

Formal reports were received concerning a total of 1932 cases tested for resistance to rifampicin. Mutations known to confer rifampicin resistance were identified in 74 leprosy cases showing a 3.8% global rifampicin resistance rate. When distinguishing between relapse (*n* = 1143) and new (*n* = 789) cases, rifampicin resistance was detected in 58 relapse cases (5.1% secondary resistance) and 16 new cases (2.0% primary resistance). Results per country are summarized in Table 1 and detailed per year and per country in the Supplementary material (Table S1).

**Fig. 1:**
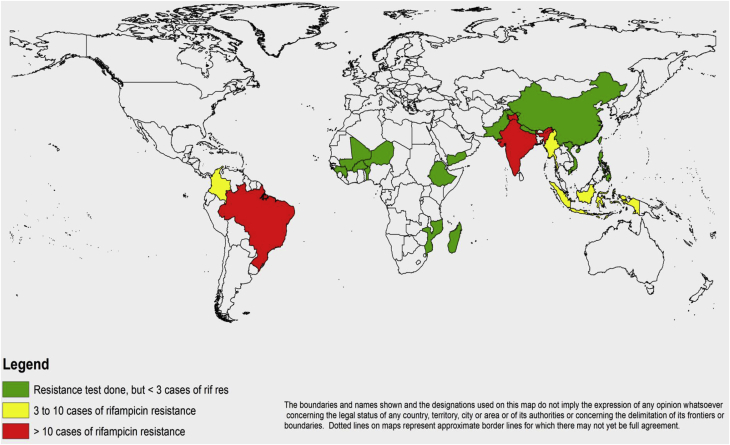
Map of countries reporting rifampicin resistance in leprosy between 2009 and 2015. Countries that reported more than ten rifampicin-resistance cases are coloured in red, those reporting between three and ten are coloured in yellow and those reporting fewer than three cases are shown in green.

As shown in Table 1 and Fig. 1, rifampicin resistance was observed in 12 countries with three of them (India, Brazil and Colombia) reporting more than five rifampicin-resistant cases over the study period. Rifampicin resistance was observed not only in relapse cases (10/18 countries testing) but in new cases (5/13 countries). Although no resistant case was observed in six countries, the number of samples tested was very low in Pakistan, Ethiopia and Burkina Faso because they stopped the surveillance for several years (see Supplementary material, Table S1). Resistance was observed in all four WHO world regions that participated in the network with 10.5% (41/390) observed in the American region, 1.8% (23/560) in the South-East Asia region (SEAR), 1.1% (5/441) in the African region (AFR), 0.7% in the Western Pacific region (WPR) (3/415) and one case (5.9%, 1/17) in the Eastern Mediterranean region (EMR) where only Yemen participated with a low number of cases tested). AMR data was obtained from the two major endemic countries, Brazil and India that tested 353 (321 relapse, 32 new) and 382 (284 relapse, 98 new) leprosy cases, respectively. Brazil reported twice as many rifampicin-resistant cases as India (32 versus 18 cases, 9.0% and 4.7% resistance rate). A twofold difference was also observed between the two countries when distinguishing new and relapse cases: 15.6% (5/32) primary resistance rate in Brazil compared with 8.2% (8/98) in India, and 8.4% (27/321) secondary resistance rate in Brazil compared with 3.5% (10/284) in India.

The rifampicin resistance-rates varied during the period of study with no clear increasing trend although the number of cases tested increased: 3/135 (2.2%) in 2010, 16/170 (9.4%) in 2011, 15/190 (4.3%) in 2012, 15/348 (4.3%) in 2013, 12/417 (2.9%) in 2014 and 10/400 (2.5%) in 2015.

### Primary and secondary resistance to dapsone and emergence of ofloxacin resistance

Results of molecular testing were obtained for *folP1*, i.e. detecting dapsone resistance, in 1639 patients (762 relapse and 877 new cases) and for *gyrA*, i.e. ofloxacin resistance, for 1581 patients (748 relapse and 833 new cases). The results are detailed per country in Table 2. Dapsone resistance was found in 87 cases (5.3% resistance rate) with secondary and primary resistance rates of 6.8% (52/762) and 4.0% (35/880), respectively. Ofloxacin resistance was found in 21 cases (1.3% resistance rate) with rates for relapse (13/748, 1.7%) and new cases (1.0%), not significantly different.

**Table 2:** Results of surveillance for dapsone (DDS) and ofloxacin (OFL) resistance (R) detailed per country for the study period 2009–15

Country	Surveillance of dapsone resistance						Surveillance of ofloxacin resistance					
	Relapse^a^		New^c^		All cases		Relapse^a^		New^c^		All cases	
	No. of cases	DDS-R^b^	No. of cases	DDS-R^d^	No. of cases	DDS-R (%)	No. of cases	OFL-R	No. of cases	OFL-R	No. of cases	OFL-R (%)
Benin	0	0	85	4	85	4 (4.7)	0	0	85	1	85	1
Brazil	187	25	26	2	213	27 (12.7)	154	3	23	0	177	3
Burkina Faso	2	0	0	0	2	0	2	0	0	0	2	0
China	61	6	55	2	116	8 (6.9)	61	0	55	0	116	0
Colombia	—	—	—	—	—	—	—	—	—	—	—	—
Ethiopia	1	0	27	0	28	0	1	0	27	0	28	0
Guinea	1	0	23	4	24	4 (16.7)	1	0	23	0	24	0
India	241	15	104	7	345	22 (6.4)	242	10	139	7	381	17
Indonesia	0	0	70	3	70	3 (4.3)	—	—	—	—	—	
Mali	20	1	115	2	135	3 (2.2)	20	0	115	0	135	0
Madagascar	13	0	103	1	116	1 (0.9)	13	0	103	0	116	0
Mozambique	5	0	0	0	5	0	7	0	0	0	7	0
Myanmar	106	3	2	0	108	3 (2.8)	106	0	2	0	108	0
Nepal	39	0	12	0	51	0	54	0	10	0	64	0
Niger	17	0	47	1	64	1 (1.6)	18	0	47	0	65	0
Philippines	43	0	140	4	183	4 (2.2)	43	0	140	0	183	0
Vietnam	13	2	62	5	75	7 (9.3)	13	0	62	0	75	0
Pakistan	5	0	0	0	5	0	5	0	0	0	5	0
Yemen	8	0	6	0	14	0	8	0	2	0	10	0
Totals	762	52	877	35	1639	87 (5.3)	748	13	833	8	1581	21

—, not tested.

a Number of relapse leprosy cases for which result was obtained.

b Number of resistant cases among relapse cases.

c Number of new leprosy cases for which result was obtained.

d Number of resistant cases among new cases.

### Mono-drug and multi-drug resistance

Among the 1932 cases studied, 154 (8.0%) *M. leprae* strains were found overall with mutations conferring resistance showing 182 resistance traits: 74 for rifampicin, 87 for dapsone and 21 for ofloxacin. Total resistance case numbers can be hypothesized with regard to the number of cases tested versus the total number of cases diagnosed per country (see Supplementary material, Table S2).

Twenty cases had rifampicin and dapsone resistance, while four had ofloxacin and dapsone resistance, but no cases, during this period of collection, were resistant to both rifampicin and ofloxacin or resistant to the three drugs. Cases with resistance to more than one drug were identified in Brazil, India and Indonesia (rifampicin plus dapsone resistance), and in Brazil and India (dapsone plus ofloxacin resistance).

## Discussion

This is the first report presenting structured data on AMR in leprosy and obtained from the main endemic countries globally over a period of 7 years. We detailed the results on rifampicin resistance, because it hampers the efficacy of the WHO standardized MDT treatment. Rifampicin-resistant cases were surprisingly present, even at low rate, in all continents and WHO regions, not only in relapse but also in new cases. Overall, the 8% resistance rate shows that the current threat to treatment is low, but it gives a baseline for future AMR surveillance.

Previous studies on AMR in leprosy have been restricted to one country or sentinel site [25], [26], [34], [35] or one national laboratory [11], [16]. In studies that distinguished relapse and new cases, resistance rates were higher in relapses, as in our study [25], [35]. However, most reports involved a low number of cases and were not structured over several years. Additionally, the centres usually did not represent nationwide data and were more research oriented. Resistance to more than one antibiotic has been already observed including resistance to the three drugs on which we focused, i.e. dapsone, rifampicin and ofloxacin [10], [11]. Our data confirmed that such resistance exists at a global level and produced basic resistance rates for the main antileprosy agents.

The strengths of this study are (a) that it generated data on AMR in leprosy over a consistent period of time from 19 endemic countries for both new cases and relapses, and (b) the successful partnership between WHO, national programmes, non-governmental organizations and expert laboratories over 7 years without much funding. There were some weaknesses, mostly due to organizational and data management issues. In addition, the use of molecular detection methods meant that phenotypic resistance could not be verified for newly described mutations. Only a handful of laboratories still perform mouse foot-pad testing, and rarely as a routine practice. In general, each sample was tested for the three resistance genes but PCR was occasionally unsuccessful for one or two of the genes, resulting in different case denominators for dapsone, rifampicin and ofloxacin resistance. In the first years of the surveillance, up to 50% of the cases tested did not give a positive PCR result [23]. This was mostly due to low bacillary loads as previously shown [16], justifying the subsequent exclusion of paucibacillary cases. Some of the expert laboratories tried out different techniques to increase the test efficiency over the study period. Although this could be viewed as a methodological weakness, satisfactory results of quality control studies and former validation of the techniques assured the quality of the AMR data.

It should be noted that we calculated the resistance rates from a very limited proportion of the total new and relapse cases of leprosy reported globally during the 7 years of the study (see Supplementary material, Table S2). The lack of a strong information system paired with the testing, might have led to a biased selection of ‘new cases’ that were taking MDT treatment for the first time but with limited or partial clinical response, which seems to apply to the cases tested in Brazil during the early years of the study. The selection of more severe or complex cases might also have resulted from the fact that most of the testing laboratories in India and Brazil are located in secondary/tertiary referral level centres, possibly leading to artificially high AMR rates. Lastly, the lack of information regarding the clinical outcomes of the patients with proven resistance testifies to the ‘disjunction’ between the laboratory and the national programme, as, until the launch of the new global strategy, this activity has not been seen as part of the routine programme activities [36].

Within the limited surveillance coverage, we highlight the potential risk of resistance to effective MDT, especially in the highest burden countries Brazil and India, where the rate of resistance is becoming noteworthy while acknowledging the limitation that in those two countries a high number of samples were tested. Because rifampicin resistance was found in new cases of leprosy, it may relate to individual medical practices and antibiotic usage [37]. This was confirmed by the occurrence of ofloxacin resistance although ofloxacin and other fluoroquinolones are not used nor recommended for the first-line treatment of leprosy.

This study reveals a potential problem that the leprosy scientific community did not foresee, and prompts the establishment of an enhanced and ‘proper’ surveillance system. For these reasons, AMR monitoring is now mentioned under the core areas of intervention in the Global Leprosy Strategy 2016–2020 ‘Accelerating towards a leprosy-free world’ [38] and WHO has recently released an updated guide on surveillance of antimicrobial resistance in leprosy [39]. In addition, it provides evidence that robust surveillance systems of AMR should be developed for all communicable diseases including leprosy [40], [41].

## Transparency declaration

No specific conflict of interest.

## Funding

Logistics, materials and meetings benefit from annual grants from ministries of health from all countries and from non-governmental agencies supporting leprosy, including Fondation Raoul Follereau, American Leprosy Missions, National Hansen's Disease Programs, Sasakawa Memorial Health Foundation, Damien Foundation and Lepra. Specific grants were also used to support the study: Swiss National Science Foundation grant IZRJZ3_164174.

## Author contributions

VP, VP, MM, EC, PS, STC and LL were responsible for conception and design of the study. All authors collected the material and data. EC, MM, STC, MK, PS, PSR, DW, ML, NC, WH, DH and VV were responsible for the laboratory procedures. PS, UDG, SA, AI, RCJ, BC, VKP, EAWDC, VRRP and LG were responsible for the logistics and organization of the network. PS, LG, STC and EC interpreted the data and PS, EC and LL wrote the main part of the paper. All authors drafted or revised and approved the final version and all authors participated in annual meetings, and presented and discussed the data.

## Appendix A Supplementary data

mmc1

mmc2

Fig. S1 Substitutions involved in antimicrobial resistance of *Mycobacterium leprae* for Rifampicin (*RpoB*), dapsone (*FolP1*) and ofloxacin (*GyrA*) resistance.
