# Supplementary material for: Antimicrobial resistance in leprosy: results of the first prospective open survey conducted by a WHO surveillance network for the period 2009–15
Source: Clin Microbiol Infect. 2018 Dec;24(12):1305–10. doi: 10.1016/j.cmi.2018.02.022 (PMC6286419; doi:10.1016/j.cmi.2018.02.022)
Supplement: mmc1 [file mmc1.docx]

**Supplementary Table 1** Detailed results of leprosy cases tested for rifampicin resistance and number of resistant cases by country by year

| **Country** | **Before 2009** | | **2009** | | **2010** | | | | **2011** | | | | **2012** | | | | **2013** | | | | **2014** | | | | **2015** | | | | **Total** | | | | |
| --- | --- | --- | --- | --- | --- | --- | --- | --- | --- | --- | --- | --- | --- | --- | --- | --- | --- | --- | --- | --- | --- | --- | --- | --- | --- | --- | --- | --- | --- | --- | --- | --- | --- |
|  | a | b | a | b | a | b | c | d | a | b | c | d | a | b | c | d | a | b | c | d | a | b | c | d | a | b | c | d | a | b | c | d |  |
| Benin |  |  |  |  |  |  | 2 | 0 |  |  | 2 | 0 |  |  | 2 | 0 |  |  | 30 | 0 |  |  | 22 | 1 |  |  | 25 | 0 | - | - | 83 | 1 |  |
| Brazil |  |  | 135 | 4 | 27 | 2 |  |  | 34 | 10 | 6 | 3 | 21 | 0 | 2 | 0 | 19 | 4 | 5 | 2 | 36 | 3 | 12 | 0 | 49 | 4 | 7 | 0 | 321 | 27 | 32 | 5 |  |
| Burkina Faso |  |  |  |  |  |  |  |  | 2 | 0 |  |  |  |  |  |  |  |  |  |  |  |  |  |  |  |  |  |  | 2 | 0 | - | - |  |
| China |  |  | 10 | 0 | 8 | 0 | 7 | 0 | 6 | 1 | 5 | 0 | 8 | 0 | 8 | 0 | 17 | 0 | 9 | 0 | 11 | 0 | 9 | 0 | 10 | 0 | 17 | 0 | 70 | 1 | 55 | 0 |  |
| Colombia | 28 | 2 | 6 | 4 | 2 | 1 |  |  | 1 | 1 |  |  |  |  |  |  |  |  |  |  |  |  |  |  |  |  |  |  | 37 | 9 | - | - |  |
| Ethiopia |  |  |  |  |  |  |  |  | 1 | 0 |  |  |  |  |  |  |  |  |  |  |  |  |  |  |  |  | 27 | 0 | 1 | 0 | 27 | 0 |  |
| Guinea |  |  |  |  |  |  |  |  |  |  |  |  |  |  | 1 | 0 |  |  | 2 | 0 | 1 | 0 | 18 | 1 |  |  | 1 | 0 | 1 | 0 | 22 | 1 |  |
| India |  |  | 27 | 0 | 31 | 0 |  |  | 32 | 0 | 12 | 1 | 40 | 0 | 17 | 1 | 72 | 4 | 28 | 2 | 37 | 3 | 5 | 1 | 45 | 3 | 36 | 3 | 284 | 10 | 98 | 8 |  |
| Indonesia | 21 | 2 |  |  |  |  |  |  |  |  |  |  |  |  |  |  |  |  |  |  | 70^e^ | 1 |  |  |  |  |  |  | 91 | 3 | - | - |  |
| Madagascar |  |  |  |  |  |  |  |  |  |  | 3 | 0 | 4 | 0 | 3 |  | 4 | 0 | 28 | 1 | 4 | 0 | 47 | 0 | 1 | 0 | 24 | 0 | 13 | 0 | 105 | 1 |  |
| Mali |  |  |  |  |  |  |  |  | 3 | 0 |  |  | 5 | 0 | 36 | 0 | 5 | 0 | 34 | 0 | 6 | 0 | 31 | 0 | 1 | 0 | 14 | 0 | 20 | 0 | 115 | 0 |  |
| Mozambique |  |  |  |  |  |  |  |  |  |  |  |  |  |  |  |  | 5 | 1 |  |  |  |  |  |  |  |  |  |  | 5 | 1 | - | - |  |
| Myanmar | 10 | 2 | 23 | 1 | 21 | 0 |  |  | 12 | 0 |  |  | 15 | 0 |  |  | 19 | 0 |  |  | 19 | 1 |  |  | 20 | 0 |  |  | 139 | 4 | - | - |  |
| Nepal |  |  |  |  | 5 | 0 | 8 | 0 | 6 | 0 | 4 | 0 | 3 | 0 | 1 | 0 | 13 | 0 | 0 | 0 | 12 | 0 | 0 | 0 | 17 | 0 | 0 | 0 | 56 | 1 | 13 | 0 |  |
| Niger |  |  |  |  |  |  |  |  |  |  |  |  |  |  |  |  | 8 | 1 | 3 | 0 | 2 | 0 | 13 | 0 | 2 | 0 | 19 | 0 | 12 | 1 | 35 | 0 |  |
| Pakistan |  |  |  |  | 8 | 0 |  |  | 1 | 0 |  |  |  |  |  |  |  |  |  |  |  |  |  |  |  |  |  |  | 9 | 0 | - | - |  |
| Philippines |  |  |  |  | 7 | 0 |  |  | 9 | 0 |  |  | 9 | 0 | 10 | 0 | 6 | 0 | 15 | 0 | 8 | 0 | 45 | 0 | 4 | 0 | 70 | 0 | 43 | 0 | 140 | 0 |  |
| Vietnam |  |  | 12 | 0 | 6 | 0 |  |  | 2 | 0 | 29 | 0 | 1 | 0 | 4 | 0 | 1 | 0 | 17 | 0 | 3 | 0 | 4 | 0 | 3 | 0 | 4 | 0 | 28 | 0 | 58 | 0 |  |
| Yemen |  |  |  |  | 3 | 0 |  |  |  |  |  |  |  |  |  |  | 2 | 0 | 6 | 0 | 2 | 1 |  |  | 4 | 0 |  |  | 11 | 1 | 6 | 0 |  |
| Total | 59 | 6 | 213 | 9 | 118 | 3 | 17 | 0 | 109 | 12 | 61 | 4 | 106 | 0 | 84 | 1 | 171 | 10 | 177 | 5 | 211 | 9 | 206 | 3 | 156 | 7 | 244 | 3 | 1143 | 58 | 789 | 16 |  |

-, not tested

a, number of relapse cases

b, rifampicin-resistant cases among relapse cases

c, number of new cases

d, rifampicin-resistant cases among new cases

e,70 cases in 2013-15 not differentiated among relapse and new cases
