# Supplementary material for: Antimicrobial resistance in leprosy: results of the first prospective open survey conducted by a WHO surveillance network for the period 2009–15
Source: Clin Microbiol Infect. 2018 Dec;24(12):1305–10. doi: 10.1016/j.cmi.2018.02.022 (PMC6286419; doi:10.1016/j.cmi.2018.02.022)
Supplement: mmc2 [file mmc2.docx]

**Supplementary Table 2.** Number of new cases and relapse cases of leprosy notified by the countries involved in the network for surveillance of drug resistance from 2009 to 2015

| Country (WHO region) | New cases of leprosy | | | | | | | Relapse cases of leprosy | | | | | | |
| --- | --- | --- | --- | --- | --- | --- | --- | --- | --- | --- | --- | --- | --- | --- |
|  | 2009 | 2010 | 2011 | 2012 | 2013 | 2014 | 2015 | 2009 | 2010 | 2011 | 2012 | 2013 | 2014 | 2015 |
| Benin (AFR) | 248 | 227 | 246 | 243 | 254 | 192 | 174 | 0 | 0 | 0 | 0 | 0 | 0 | 0 |
| Brazil (AMR) | 37160 | 34894 | 33955 | 33303 | 31044 | 31064 | 26395 | 1483 | NR | 1498 | 1709 | 1603 | 0 | 1452 |
| Burkina Faso (AFR) | 412 | 320 | NR | 313 | 253 | 208 | 187 | 12 | 6 | NR | 0 | 0 | 0 | 0 |
| China (WPR) | 1597 | 1324 | 1144 | 1206 | 924 | 823 | 678 | 148 | 96 | 84 | 84 |  | 53 | 42 |
| Colombia (AMR) | 468 | 295 | 434 | 392 | 430 | 423 | 349 | 19 | 55 | 58 | 50 | 58 | 30 | 25 |
| Ethiopia (AFR) | 4417 | 4430 | NR | 3776 | 4374 | 3758 | 3970 | 312 | 357 | NR | 194 | 0 | NR | NR |
| Guinea (AFR) | 636 | 549 | 498 | 438 | 387 | 241 | 184 | 63 | NR | 0 | 0 | 0 | 0 | 0 |
| India (SEAR) | 133717 | 126800 | 127295 | 134752 | 126913 | 125785 | 127326 | 670 | 907 | 690 | 697 | 919 | 671 | 459 |
| Indonesia (WPR) | 17260 | 17012 | 16099 | 18994 | 16856 | 17025 | 17202 | 48 | 147 | 164 | 194 | 187 | 0 | 526 |
| Madagascar (AFR) | 1572 | 1520 | 1577 | 1474 | 1569 | 1617 | 1487 | 0 | 8 | 5 | 17 | 18 | 27 | 17 |
| Mali (AFR) | 346 | 363 | 226 | 228 | 176 | 259 | 222 | 0 | 0 | 0 | 0 | 0 | 0 | 0 |
| Mozambique (AFR) | 1191 | 1207 | 1097 | 758 | NR | NR | 1355 | NR | 0 | 0 | 4 | NR | NR | 2 |
| Myanmar (SEAR) | 3147 | 2936 | 3082 | 3013 | 2950 | 2877 | 2571 | 25 | 31 | 18 | 16 | 11 | 7 | 17 |
| Nepal (SEAR) | 4394 | 3118 | 3184 | 3492 | 3225 | 3046 | 2751 | 23 | 96 | 20 | 17 | 14 | 17 | 22 |
| Niger (AFR) | 555 | 447 | 364 | 464 | 424 | 403 | 378 | 1 | 0 | 0 | 0 | 0 | 0 | 0 |
| Pakistan (EMR) | 527 | 396 | 429 | 377 | 431 | 501 | 446 | 11 | 15 | 10 | 14 | 11 | 11 | 14 |
| Philippines (WPR) | 1795 | 2041 | 1818 | 2150 | 1729 | 1655 | 1617 | 12 | 18 | 85 | 65 | 53 | 57 | 45 |
| Viet nam (WPR) | 413 | 359 | 748 | 295 | 260 | 187 | 178 | 9 | 5 | 7 | 8 | 14 | 11 | 6 |
| Yemen (EMR) | 387 | 339 | 299 | 392 | 383 | 413 | 225 | 6 | 3 | 3 | 4 | 2 | 6 | 4 |
| Total | 210242 | 198577 | 192495 | 206060 | 192582 | 190477 | 187695 | 2842 | 1744 | 2642 | 3073 | 2890 | 890 | 2631 |

NR, not reported; AFR, African region; AMR, American region; WPR, Western pacific region; SEAR, South-East Asia region.
