# Supplementary material for: Antimicrobial resistance in leprosy: results of the first prospective open survey conducted by a WHO surveillance network for the period 2009–15
Source: Clin Microbiol Infect. 2018 Dec;24(12):1305–10. doi: 10.1016/j.cmi.2018.02.022 (PMC6286419; doi:10.1016/j.cmi.2018.02.022)
Supplement: Fig. S1 — Substitutions involved in antimicrobial resistance of Mycobacterium leprae for Rifampicin (RpoB), dapsone (FolP1) and ofloxacin (GyrA) resistance. [file mmc3.docx]

RpoB substitutions associated with rifampicin resistance in *Mycobacterium leprae*

| A |  | 508 |  | 511 |  | 513 |  | 516 |  |  | 526 | 531 | 533 |
| --- | --- | --- | --- | --- | --- | --- | --- | --- | --- | --- | --- | --- | --- |
| B |  | 402 |  | 405 |  | 407 |  | 410 |  |  | 420 | 425 | 427 |
| C | **432** | **433** | **434** | **436** | **437** | **438** | **439** | **441** | **442** | **448** | **451** | **456** | **458** |
| D | **Gly** | **Thr** | **Ser** | **Leu** | **Ser** | **Gln** | **Phe** | **Asp** | **Gln** | **Gly** | **His** | **Ser** | **Leu** |
| E | *Ser*? | *Ile* | *Gly* | *Pro* | *Val* | VAL | *Leu* | *Tyr* | *His* | *Cys* | ASP | LEU | VAL |
|  |  |  | *Cys* |  |  |  |  | *Asn* |  |  | TYR | MET | *Pro?* |
|  |  |  |  |  |  | insertion |  |  |  |  |  | PHE |  |
|  |  |  |  |  |  |  |  |  |  |  |  | *Trp* |  |
|  |  |  |  |  |  |  |  |  |  |  |  | *Val* |  |

FolP1 substitutions associated dapsone resistance in *Mycobacterium leprae*

| A | 62 | 63 | 64 |
| --- | --- | --- | --- |
| **C** | **53** | **54** | **55** |
| **D** | **Thr** | **Arg** | **Pro** |
| E | ILE |  | ARG |
|  | ARG |  | LEU |
|  | ALA |  |  |

GyrA substitutions associated with ofloxacin resistance in *Mycobacterium leprae*

| A | 81 | 82 | 83 | 84 | 85 | 86 | 87 | 88 |
| --- | --- | --- | --- | --- | --- | --- | --- | --- |
| **C** | **89** | **90** | **91** | **92** | **93** | **94** | **95** | **96** |
| **D** | **Gly** | **Asp** | **Ala** | **Ser** | **Ile** | **Tyr** | **Asp** | **Thr** |
| E | CYS |  | VAL |  |  |  |  |  |
